# Juçara pulp supplementation improves glucose tolerance in mice

**DOI:** 10.1186/s13098-015-0122-4

**Published:** 2016-01-22

**Authors:** L. M. Oyama, F. P. Silva, J. Carnier, D. A. de Miranda, A. B. Santamarina, E. B. Ribeiro, C. M. Oller do Nascimento, V. V. de Rosso

**Affiliations:** Departamento de Fisiologia, Escola Paulista de Medicina-Universidade Federal de São Paulo, Rua Botucatu, 862-Vila Clementino, São Paulo, SP Brazil; Departamento de Biociências, Instituto de Saúde e Sociedade, Universidade Federal de São Paulo, Santos, São Paulo Brazil

**Keywords:** Juçara, Euterpe edulis Mart, Obesity, Inflammation, Mice, Anthocyanins

## Abstract

**Background:**

The consumption of hyperlipidic and hypercaloric diet is considered a major factor to promote obesity and the consumption of food with antioxidant properties, like Juçara (*Euterpe edulis* Mart), could be a tool to prevent the deleterious effect of high white adipose deposition. The aim of the present study was to evaluate the effect of administration of juçara pulp in mice fed a high-fat, high-calorie diet on glucose tolerance and adipose tissue inflammatory status.

**Methods:**

Mice were distributed into the following groups: control diet; control diet plus 0.5 % of juçara; control diet plus 2 % of juçara; hypercaloric and hyperlipidic diet; hypercaloric and hyperlipidic diet plus 0.5 % of juçara and hypercaloric and hyperlipidic diet plus 2 % of juçara. Treatments started when mice were 8 weeks old and carried on for a total period of 10 weeks. The serum glucose, triacylglycerol, total cholesterol, insulin, adiponectin, lipopolysaccharides and free fatty acids concentrations were measured. Oral glucose tolerance test was performed. TNF-α, IL-6, and IL-10 protein level were determined by ELISA on mesenteric and epididymal white adipose tissues. Determination of catalase activity was realized in the same tissues. Data were analysed using one-way analysis of variance and post hoc analysis was performed with the Tukey’s test.

**Results:**

The addition of 0.5 % juçara improved glycemic response in animals that consumed normocaloric as well as hypercaloric and hyperlipidic diets (HC). Supplementation with 0.5 and 2 % did not change the body composition of animals that received the HC diet; however, the animals fed the normocaloric diet with 2 % juçara gained body mass. An intake of 2 % juçara in the HC diet promoted a reduction of catalase activity and IL-10 level in epididymal adipose tissue.

**Conclusions:**

These results suggest that with the administration of 0.5 % juçara, the beneficial effects of polyphenols overcome the deleterious effects of macronutrient composition of juçara, whereas with the administration of 2 % juçara promotes damage by the composition of the fruit and overshadows the beneficial effects of polyphenols on glucose metabolism. On the other hand, higher juçara supplementation improves the inflammatory status targeted by the HC diet.

## Background

Obesity is now recognized as a low-grade chronic inflammatory disease that is linked to health problems including cardiovascular diseases, cancer, and type 2 diabetes [1–4]. In Brazil, in 2007, approximately 72 % of deaths were associated with chronic diseases, including cardiovascular diseases and diabetes [5]. Environmental factors such as the intake of inappropriate meals with high caloric values and low micronutrients and physical inactivity are the factors that contribute most to the development of obesity in humans [6].

The chronic subclinical inflammatory condition of obesity is characterized by the abnormal production of cytokines and proinflammatory adipokines, increase in acute-phase protein, and activation of inflammatory signaling pathways [7]. Diet can positively or negatively influence the metabolic control of glucose homeostasis and alter the production of adipokines involved in insulin sensitivity, such as adiponectin and TNF-α [8, 9].

Foods rich in antioxidants are receiving attention with the purpose of mitigating, by means of their bioactive compounds, the deleterious effects of the modern fat- and sugar-rich diet. Açaí (*Euterpe oleracea* Mart) is an Amazon fruit widely consumed in Brazil and in different parts of the world. The pulp of açaí contains high levels of antioxidants and is a source of antioxidants in the Brazilian diet [10–12].

A fruit very similar to açaí and yet little studied is the juçara (*Euterpe edulis* Mart). The juçara palm is native to the Atlantic Forest and is distributed in Brazil from the state of Bahia to the north of the state of Rio Grande do Sul [13]. The palmetto of this palm is one of the most abundant and valuable non-timber products in the Atlantic forest [14]; however, its extraction leads to death of the palm. In addition to its economic value, this palm is ecologically important in the forest ecosystem [15]. However, its fruit is still consumed less than that of açaí [16].

Juçara contains high levels of phenolic compounds and anthocyanins [12], bioactive compounds linked with antioxidant activity. Among anthocyanins, the majority are cyanidin-3-glucoside and cyanidin-3-rutinoside [16, 17]. The profiles of fatty acids have been evaluated, revealing the prevalence of monounsaturated fatty acids, mainly oleic acid [18, 19].

Few studies have investigated the effects of juçara in vivo. In animals, juçara exerted a beneficial effect in the offspring of mothers who consumed the fruit in the perinatal period. There was an improvement in the lipid profile, glucose concentration, body composition, and intestinal microbiota and a reduction in low-grade inflammation in the colon of 21-day-old puppies [20]. In contrast, supplementation with 2 % juçara of the diet of *ApoE*^−^*/*^−^ mice for 12 weeks did not affect hepatic oxidation or the inflammatory profile of these animals [21].

In view of the proximity of the supply and the need to preserve this palm tree, which also contributes to the local economy, in the Atlantic Forest ecosystem, it is desirable to characterize the in vivo the metabolic effects of this fruit to stimulate consumption. The aim of the present study was to evaluate the effect of administration of juçara pulp to mice fed with high fat and high caloric diet on glucose tolerance and adipose tissue inflammatory status.

## Methods

### Animals and treatment

The Experimental Research Committee of the Universidade Federal de São Paulo approved all procedures for the care of the animals used in this study (CEUA 497155). Mice were kept under controlled conditions of light (12-h: 12-h light–dark cycle with lights on at 6:00 a.m.) and temperature (24 ± 1 °C) at 60 ± 5 % humidity and with free access to food and water. Sixty six four-week-old male Swiss mice were purchased from Biotério Central da Universidade Estadual de Campinas—CEMIB and given 1 week of acclimation in our facility. After that, mice were distributed semi-randomly into the following groups (10–14 animals for each treatment): control diet (C group); control diet plus 0.5 % of juçara (CJ0.5 % group); control diet plus 2 % of juçara (CJ2 % group); high fat and high caloric diet (HC group); high fat and high caloric diet plus 0.5 % of juçara (HJ0.5 %) and high fat and high caloric diet plus 2 % of juçara (HJ2 %). From 5 to 7 week-old the animals received a commercial standard diet (Nuvilab, Brasil). Treatments started when mice were 8 weeks old and carried on for a total period of 10 weeks. The controls diets (normocaloric and normolipidic) were prepared according to the recommendations of the American Institute of Nutrition (AIN-93) [22] with energy value of 3.802,8 Kcal/kg, 75.8 % of which by carbohydrates, 14.7 % by proteins and 9.5 % by lipids (soybean oil). The high fat and high caloric diets were adapted from AIN-93 with energy value of 5100 Kcal/kg, 38.8 % of which by lipids (lard and soybean oil), 47.1 % by carbohydrates and 14.1 % by proteins. The diets prepared with juçara were submitted to adjustments to maintain the same final caloric value. The CJ0.5 % and HJ0.5 % diets were prepared by adding 5 g/kg and CJ2 % and HJ2 % diets were prepared by adding 20 g/kg of juçara freeze-dried powder to each diet. Juçara pulp (*Euterpe edulis* Mart.) was obtained from the agroecological Project Juçara/IPEMA-Institute of Permaculture and Ecovillages of the Atlantic (Ubatuba, SP, Brazil) and then freeze-dried to powder using a lyophilizer. All treatments were performed using same lot. Diets were then stored at −20 °C. The phenolic compounds and anthocyanin contents of the juçara pulp were previously analyzed [17]. The centesimal composition of the diets is presented in Table 1.

**Table 1:** Composition of the control diet (C), control diet supplemented with 0.5 % freeze-dried Juçara (CJ0.5 %), control diet supplemented with 2 % freeze-dried Juçara (CJ2 %), hypercaloric and hyperlipidic diet (HC), hypercaloric and hyperlipidic diet supplemented with 0.5 % freeze-dried Juçara (HJ0.5 %) and hypercaloric and hyperlipidic diet supplemented with 2 % freeze-dried Juçara (HJ 2 %)

Ingredients (g/kg of diet)	C	CJ0.5 %	CJ2 %	HC	HJ0.5 %	HJ2 %
Cornstarch	720.7	717.85	709.3	450	450	450
Sucrose	–	–	–	150	147.15	138.6
Casein	140	140	140	180	180	180
Soybean oil	40	38.25	33	40	38.25	33
Lard	–	–	–	180	180	180
Cellulose	50	48.39	43.6	–	–	–
Vitamin mix	10	10	10	10	10	10
Mineral mix	35	35	35	35	35	35
L-cystine	1.8	1.8	1.8	1.8	1.8	1.8
Choline bitartrate	2.5	2.5	2.5	2.5	2.5	2.5
BHT	0.008	0.008	0.008	0.008	0.008	0.008
Freeze-dried Juçara	0	5	20	0	5	20

### Oral glucose tolerance test (OGTT)

The OGTT was performed 7 days before euthanasia, from 8:00 to 11:00. After an overnight fast, basal glycemia was measured from the tail vein using a glucometer (Accu-Check, Roche Diagnóstica Brasil Ltda., São Paulo, SP, Brazil). Immediately after the first measure, glucose solution (2 g/kg) was administrated by gavage. Blood samples were collected after 15, 30, 45, 60, and 120 min to obtain the area under the glycemic curve.

### Experimental procedures

At the end of the experimental period, animals were fasted for 12 h overnight prior to being euthanatized by decapitation. Trunk blood was collected and immediately centrifuged (1125 g/15 min at 4 °C). Serum was separated and stored at −80 °C for later biochemical and hormonal determination. The white adipose tissue depots, retroperitoneal (RET), mesenteric (MES), and epididymal (EPI), were dissected, weighed, immediately frozen in liquid nitrogen, and stored at −80 °C.

### Serum analyses

The serum glucose, triacylglycerol (TG) and total cholesterol concentrations were measured using a commercial enzymatic colorimetric kit (Labtest, Lagoa Santa, MG, Brazil). The insulin and adiponectin serum concentrations were quantified using specific enzyme-linked immunosorbent assay (ELISA) kits (R&D Systems and Millipore). Lipopolysaccharides (LPS) and free fatty acids (FFA) were determined using a commercials kit (Lonza and Zembio, respectively).

### TNF-α, IL-6, and IL-10 protein level determined by ELISA

Following euthanasia, mesenteric and epididymal white adipose tissues were removed, homogenized into a specific total protein extraction buffer (1 % Triton X-100, 100 mm Tris–HCl (pH 7.4), 100 mm sodium pyrophosphate, 100 mm sodium fluoride, 10 mm EDTA, 10 mm sodium orthovanadate, 2.0 mm phenylmethylsulfonyl fluoride, and 0.1 mg aprotinin/mL), and centrifuged at 12,000 g for 40 min at 4 °C. The supernatant was collected and protein content was analyzed with a Bradford assay kit (Bio-Rad, Hercules, CA, USA) with bovine serum albumin (BSA) as a reference. Quantitative assessment of TNF-α, IL-6, and IL-10 proteins were carried out by ELISA (Duo Set ELISA, R&D Systems, Minneapolis, MN, USA) following the recommendations of the manufacturer.

### Determination of antioxidant enzyme activity in the mesenteric and epididymal white adipose tissues

Mesenteric and epididymal white adipose tissues fragments were homogenized in 50 mM-phosphate buffer and the resulting suspension was centrifuged at 3000 g at 4 °C for 10 min. The supernatant was used to measure enzyme activity. Catalase (CAT) activity was determined by the rate of decay of H_2_O_2_ read in a spectrophotometer (Beckman DU640) at 240 nm, as described previously [23].

### Statistical analysis

Distribution assumptions were verified by the Shapiro–Wilk test; homogeneity, by the Levene’s test. Data were standardized to log base 10 values when needed. Effects of treatment were analysed using one-way analysis of variance (ANOVA). When ANOVA showed significant differences, post hoc analysis was performed with the Tukey’s test. All data are expressed as mean ± standard error (SEM). The level of significance was p ≤ 0.05. Statistical analyses were performed using *StatsDirect*–*Statistical Software*.

## Results

### Ogtt

**Fig. 1:**
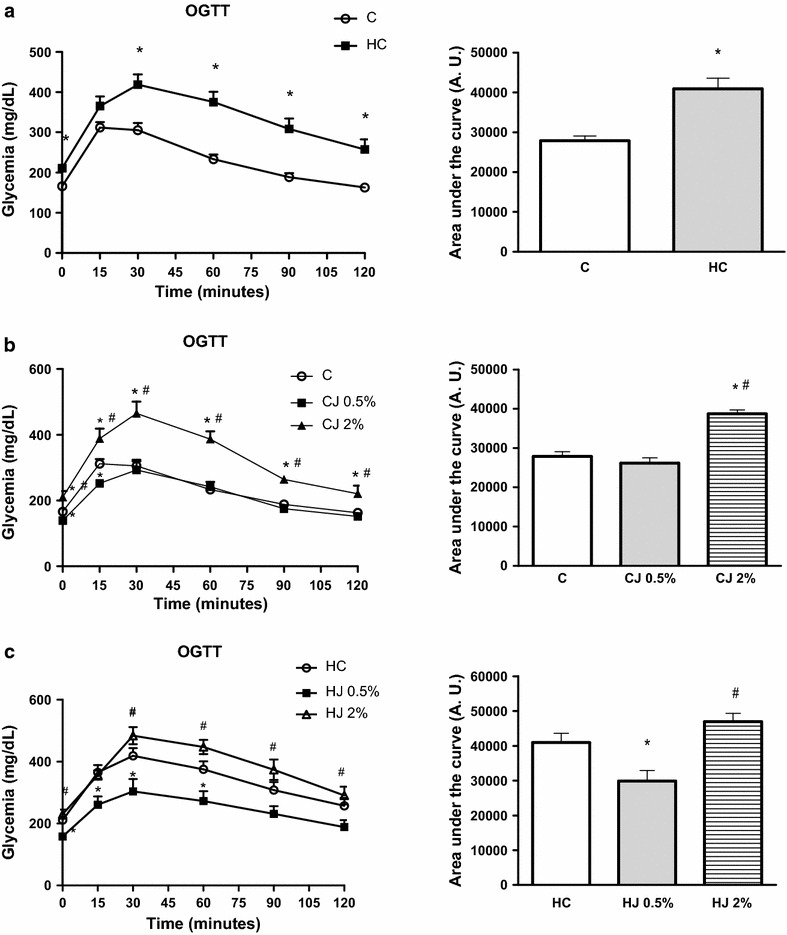
Oral glucose tolerance test (OGTT) and area under the curve (AUC) of C and HC groups (**a**); C, CJ0.5 % and CJ2 % (**b**); HC, HJ0.5 % and HJ2 % (**c**) after treatment. Glycemia in time zero (basal), 15, 30, 60, 90, and 120 min after gavage of 2 g/kg body weight of glucose. *Asterisk* significantly difference of C group (**a**, **b**) and HC group (**c**); *Bash* significantly difference of CJ 0.5 % group (**b**) and HJ 0.5 % group (**c**); p < 0.04

Figure 1a shows that the C group showed significantly lower area under the curve (AUC) and OGTT values at baseline and at 30, 60, 90, and 120 min than the HC group. Figure 1b shows that the CJ2 % group showed significantly higher values than the C and CJ0.5 % groups at all times. Only at baseline and 15 min did the CJ0.5 % group show lower values than the C group. The AUC level was higher in the CJ2 % than that in the C and CJ0.5 % groups. Among HC groups (panel C), OGTT values showed statistically lower values in the HJ0.5 % group at baseline and at 15, 30, and 60 min than those in the HC group. The HJ0.5 % group showed significantly decreased values at baseline and at 30, 60, 90, and 120 min compared with the HJ2 % group. AUC values was statistically lower in HJ0.5 % group than those in HC and HJ2 % groups.

### Body and adipose tissues weight

The HC group showed body mass gain higher than that of the C group after treatment. The hypercaloric diet promoted an increase in the relative mass of retroperitoneal and epididymal adipose tissues and adiposity compared with the control group (Table 2). The CJ2 % group showed higher body mass gain than the C and CJ0.5 % groups. Animals that received a standard diet with an additional 2 % juçara showed higher relative mass of mesenteric adipose tissue than the C and CJ0.5 % groups (Table 3). When all HC groups were compared, no statistical difference was found. No differences were observed in the relative mass of adipose tissues and adiposity between hypercaloric groups when juçara was added to the hypercaloric diet (Table 4).

**Table 2:** The relative mass of tissues (mg/g of body mass), adiposity (g) and body mass gain (g) after 10 weeks treatment of mice of C and HC groups

	C (n = 12)	HC (n = 14)
RET	0.82 ± 0.06	1.07 ± 0.06*
EPI	3.77 ± 0.34	5.01 ± 0.37*
MES	1.8 ± 0.15	2.17 ± 0.21
Adiposity	6.4 ± 0.5	8.3 ± 0.5*
Δ Body mass	10.8 ± 0.86	16 ± 1.89*

Data were expressed as mean ± SEM

* Significantly difference of C group; p < 0.05

Adiposity: Ʃ of epididymal, retroperitoneal, and mesenteric relative weight

**Table 3:** Relative mass of tissues (mg/g of body mass), adiposity (g) and body mass gain (g) after 10 weeks treatment of C, CJ0.5 % and CJ2 % groups

	C (n = 12)	CJ 0.5 % (n = 11)	CJ 2 % (n = 9)
RET	0.82 ± 0.06	0.9 ± 0.07	0.96 ± 0.05
EPI	3.77 ± 0.34	3.74 ± 0.28	4.35 ± 0.15
MES	1.8 ± 0.15	1.85 ± 0.14	2.44 ± 0.16^*,**^
Adiposity	6.4 ± 0.5	6.48 ± 0.42	7.74 ± 0.21
Δ Body mass	10.8 ± 0.86	12.4 ± 0.98	18.9 ± 2.09^*,**^

Data were expressed as mean ± SEM

Adiposity Ʃ of epididymal, retroperitoneal, and mesenteric relative weight

* Significantly difference of C group

^**^Significantly difference of CJ 0.5 % group; p < 0.03

**Table 4:** Relative mass of tissues (mg/g of body mass), adiposity (g) and body mass gain (g) after 10 weeks treatment of HC, HJ0.5 % and HJ2 % groups

	HC (n = 14)	HJ 0.5 % (n = 10)	HJ 2 % (n = 10)
RET	1.07 ± 0.06	0.98 ± 0.05	1.09 ± 0.1
EPI	5.01 ± 0.37	4.19 ± 0.21	4.68 ± 0.25
MES	2.17 ± 0.21	1.97 ± 0.15	2.13 ± 0.19
Adiposity	8.3 ± 0.5	7.15 ± 0.22	7.9 ± 0.4
Δ Body mass	16 ± 1.89	13.8 ± 1.13	14.8 ± 1.62

Data were expressed as mean ± SEM

Adiposity: Ʃ of epididymal, retroperitoneal, and mesenteric relative weight

### Serum analysis

With respect to the biochemical parameters of the C and HC groups, the HC group showed higher levels of total cholesterol but lower levels of triacylglycerol and free fatty acids than the control group. Other parameters were not statistically different (Table 5). The CJ2 % group showed higher levels of glucose than the C and CJ0.5 % groups. Total cholesterol was higher in the CJ2 % group than that in the C group, and HOMA-IR was greater than in the CJ0.5 % group. Adiponectin levels were statistically lower in the CJ0.5 % group than those in the C group (Table 6). The HJ2 % group showed higher insulin and adiponectin levels than the HJ0.5 % group (Table 7), whereas other parameters were not significantly different between groups.

**Table 5:** Biochemical parameters of C and HC groups after 10 weeks treatment

Parameters	C (n = 9–12)	HC (n = 11–14)
Total cholesterol (mg/dL)	164.7 ± 9.2	196.9 ± 12.2*
Triacylglycerol (mg/dL)	201.8 ± 12.2	159.6 ± 7.7*
Insulin (ng/mL)	1.7 ± 0.3	1.6 ± 0.3
HOMA-IR	11.5 ± 2.2	12.2 ± 2
FFA (mM)	3.4 ± 0.2	2.6 ± 0.2*
LPS (EU/mL)	3.4 ± 0.3	3.1 ± 0.1
Adiponectin (µg/mL)	6.1 ± 0.4	5.4 ± 0.6

Data were expressed as mean ± SEM

*HOMA-IR* homeostasis model assessment, *FFA* free fatty acids, *LPS* lipopolysaccharides

* Significantly difference of C group; p < 0.05

**Table 6:** Biochemical parameters of C, CJ0.5 % and CJ2 % groups after 10 weeks treatment

Parameters	C (n = 9–12)	CJ 0.5 % (n = 9–11)	CJ 2 % (n = 8–10)
Total cholesterol (mg/dL)	164.7 ± 9.2	198 ± 14.3	248 ± 19.6*
Triacylglycerol (mg/dL)	201.8 ± 12.2	198.7 ± 7.9	189.9 ± 17.9
Insulin (ng/mL)	1.7 ± 0.3	1.2 ± 0.1	1.9 ± 0.4
HOMA-IR	11.5 ± 2.2	5.7 ± 0.5	14.1 ± 2.4^**^
FFA (mM)	3.4 ± 0.2	3 ± 0.2	2.8 ± 0.4
LPS (EU/mL)	3.4 ± 0.3	2.9 ± 0.1	2.9 ± 0.1
Adiponectin (µg/mL)	6.1 ± 0.4	4.3 ± 0.3*	5.1 ± 0.6

Data were expressed as mean ± SEM

*HOMA-IR* homeostasis model assessment, *FFA* free fatty acids, *LPS* lipopolysaccharides

* Significantly difference of C group

** Significantly difference of CJ 0.5 % group; p < 0.02

**Table 7:** Biochemical parameters of HC, HJ0.5 % and HJ2 % groups after 10 weeks treatment

Parameters	HC (n = 11–14)	HJ 0.5 % (n = 9–10)	HJ 2 % (n = 9–10)
Total cholesterol (mg/dL)	196.9 ± 12.2	175.5 ± 14.2	210.9 ± 12.4
Triacylglycerol (mg/dL)	159.6 ± 7.7	145.8 ± 8.4	144.9 ± 5.6
Insulin (ng/mL)	1.6 ± 0.3	0.97 ± 0.12	3.12 ± 1.03^*^
HOMA-IR	12.2 ± 2	6.98 ± 1.16	28 ± 10.8
FFA (mM)	2.6 ± 0.2	2.27 ± 0.22	2.63 ± 0.2
LPS (EU/mL)	3.1 ± 0.1	2.84 ± 0.12	3.2 ± 0.19
Adiponectin (µg/mL)	5.4 ± 0.6	3.87 ± 0.39	6.39 ± 0.7*

Data were expressed as mean ± SEM

*HOMA-IR* homeostasis model assessment, *FFA* free fatty acids, *LPS* lipopolysaccharides

* Significantly difference of HJ 0.5 % group; p ≤ 0.04

### Concentration of IL-6, IL-10, and TNF-α in the epididymal, and mesenteric white adipose tissues and catalase activity

With respect to cytokine concentrations, animals that received hypercaloric diets showed higher levels of IL-6 and IL-10 in epididymal adipose tissue and higher levels of IL-6 in mesenteric adipose tissue than the control group (Table 8). The control diet plus 0.5 % juçara led to the higher concentrations of TNF-α, IL-6, and Il-10 in epididymal adipose tissue than in the control group, whereas the addition of 2 % juçara led to the higher levels of IL-6 in epididymal adipose tissue than in animals receiving a normal diet (Table 9). The group treated with HJ2 % showed lower levels of IL-10 in epididymal adipose tissue and of IL-6 in mesenteric adipose tissue than the HC group. The activity of catalase in epididymal adipose tissue was significantly lower in the HJ2 % group than that in the HC group (Table 10).

**Table 8:** Concentrations of epididymal and mesenteric tissue citokines (pg/mg of total protein content) and catalase activity (U CAT/mg of total protein content) of C and HC groups after 10 weeks treatment

	C (n = 4–7)	HC (n = 4–7)
EPI		
TNFα	60.3 ± 7.4	79.8 ± 8.1
IL6	41.8 ± 3.7	56.5 ± 3.8*
IL10	131 ± 21.3	216.1 ± 15.2*
Catalase	1.27 ± 0.29	1.76 ± 0.19
MES		
TNFα	28.2 ± 7.1	22.9 ± 3.2
IL6	18.2 ± 1.3	38.2 ± 6.3*
IL10	78.5 ± 15.4	92.6 ± 17.3
Catalase	1.9 ± 0.37	1.4 ± 0.09

Data were expressed as mean ± SEM

* Significantly difference of C group; p < 0.03

**Table 9:** Concentrations of epididymal and mesenteric tissue citokines (pg/mg of total protein content) and catalase activity (U CAT/mg of total protein content) of C and HC groups after 10 weeks treatment

	C	CJ 0.5 %	CJ 2 %
	(n = 4–7)	(n = 3–7)	(n = 4–7)
EPI			
TNFα	60.3 ± 7.4	108.9 ± 8.5*	87.5 ± 7.7
IL6	41.8 ± 3.7	79.9 ± 8.7*	73.1 ± 8.9*
IL10	131 ± 21.3	197.3 ± 15.2*	184 ± 13.9
Catalase	1.27 ± 0.29	1.42 ± 0.07	1.73 ± 0.32
MES			
TNFα	28.2 ± 7.1	30.7 ± 5.2	27.1 ± 2.5
IL6	18.2 ± 1.3	25.3 ± 5	25.3 ± 3.4
IL10	78.5 ± 15.4	128.9 ± 27.5	97.2 ± 6.8
Catalase	1.9 ± 0.37	1.57 ± 0.15	1.26 ± 0.17

Data were expressed as mean ± SEM

* Significantly difference of C group; p < 0.04

**Table 10:** Concentrations of epididymal and mesenteric tissue citokines (pg/mg of total protein content) and catalase activity (U CAT/mg of total protein content) of C and HC groups after 10 weeks treatment

	HC	HJ 0.5 %	HJ 2 %
	(n = 4–7)	(n = 4–7)	(n = 4–7)
EPI			
TNFα	79.8 ± 8.1	76.57 ± 15.57	66.97 ± 5.64
IL6	56.5 ± 3.8	53.36 ± 5.48	48.67 ± 2.99
IL10	216.1 ± 15.2	171.3 ± 17.7	140.1 ± 11.2*
Catalase	1.76 ± 0.19	1.27 ± 0.14	0.98 ± 0.18*
MES			
TNFα	22.9 ± 3.2	42.97 ± 11.32	24.6 ± 2
IL6	38.2 ± 6.3	24.9 ± 5.7	18.4 ± 3.25*
IL10	92.6 ± 17.3	114.7 ± 18.9	80.4 ± 6.15
Catalase	1.4 ± 0.09	1.61 ± 0.16	1.76 ± 0.25

Data were expressed as mean ± SEM

* Significantly difference of HC group; p < 0.04

## Discussion

There are few studies of juçara effects in vivo. To the best of our knowledge, this is the first investigation to evaluate the influence of juçara on glucose metabolism and inflammation according to dose. One of the findings of the present study was that supplementation with 0.5 % juçara, both in normocaloric and in hypercaloric diets, promoted an improvement in glucose metabolism. Rufino et al. (2010) [12] studied the composition of 18 Brazilian tropical exotic fruits, including açai and juçara. With respect to bioactive compounds, juçara showed the higher concentrations of vitamin C (186 mg/100 g), anthocyanins (192 mg/100 g), and phenolic compounds (755 mg GAE/100 g). Of the total anthocyanins of juçara described by De Brito et al. (2007) [16], it was possible to separate and identify six different anthocyanins. The major anthocyanins were cyanidin-3-glucoside and cyanidin-3-rutinoside. Previous studies showed that the administration of cyanidin-3-glucoside ameliorates hyperglycemia in mice, possibly owing to the increased expression of GLUT-4 and decreased expression of RBP4 [24, 25].

Adiponectin is an antiinflammatory adipokine inversely associated with insulin resistance [26]. Although CJ0.5 % showed an amelioration in glucose intolerance, the adiponectin was reduced in this group in comparison with the C group. The higher concentrations of TNF-α and IL-6 in epididymal adipose tissue may partially explain this reduction in adiponectin [2].

The hypercaloric and hyperlipidic diet was effective for promoting the development of inflammatory process in the present study. We expected that juçara supplementation would minimize and/or reverse inflammatory processes. In fact, our results suggested that supplementation with 2 % juçara in a hypercaloric and hyperlipidic diet reduced inflammation by reducing catalase activity, concentrating IL-10 in epididymal adipose tissue, and reducing IL-6 concentration in mesenteric adipose tissue, as another highlight of the present investigation. However, supplementation with 0.5 % juçara in a hypercaloric and hyperlipidic diet was not sufficient to reverse this process, showing that the bioactive compounds present in juçara, mainly phenolic compounds and in particular, anthocyanins, reduced inflammation associated with a hyperlipidic diet only when fed with 2 % amounts [16, 17].

Supplementation with 0.5 % juçara in a normocaloric diet reduced inflammation in epididymal adipose tissue after 10 weeks of treatment. As a compensatory mechanism, the same animal showed an increase of IL-10 in the same adipose tissue. Although similar results were not observed with supplementation with 2 % juçara in a normocaloric diet, an increase in the concentrations of IL-6 in epididymal adipose tissue was observed. Similarly, Karlsen et al. (2010) [27] found in a human study an increase of TNF-α concentration in subjects receiving bilberry juice, which is rich in anthocyanin, for 4 weeks. These curious results may be partially explained by a dependence of juçara effects on dose, target tissue, or target gene. The effect of supplementation with 2 % juçara of a standard diet on the inflammatory process awaits further study.

The results presented in Table 2 show that the period of treatment and diet composition were effective in inducing obesity as expected, with animals showing increased relative mass of epididymal and retroperitoneal fat deposits, adiposity, and body mass gain. Surprisingly, animals fed a normocaloric diet supplemented with 2 % juçara showed higher body mass gain during treatment, whereas the HJ0.5 % and HJ2 % groups did not show the same result. In contrast to our results, Heyman et al. (2014) [28] found that supplementation with a high concentration (20 %) of açai promoted an increase in body mass gain in mice that received a high fat diet, relative to the effect of other berries.

Besides the deleterious effects caused in body composition by the high fat diet in the HC group, some biochemical parameters were altered as well. Despite these changes, the HC group showed lower triacylglycerol and free fatty acids after treatment. These results can be explained by the higher amounts of carbohydrates in the normocaloric than in the hypercaloric diet. It may be that the high-fat diet stimulated lipoprotein lipase activity and consequently the uptake of fatty acids by adipose tissue, decreasing hepatic lipogenesis and reducing serum TG as observed in the present study [29, 30].

Unexpectedly, our results showed an increase in total cholesterol promoted by 2 % juçara supplementation of a standard diet. In a similar mouse study using supplementation with 2 % juçara of a normocaloric diet for 12 weeks, the authors found contradictory results, although the serum lipid profile was unaffected [21]. The higher levels of total cholesterol in CJ2 % can be accounted for by the composition of juçara. All diets used in the present study had the same energy content. However, juçara contains a high amount of lipids, of which the majority is monounsaturated fatty acids, followed by saturated and polyunsaturated fatty acids in lower amounts [18].

## Conclusions

These novel results show that when 0.5 % juçara is fed, the beneficial effects of polyphenols overcome the deleterious effects of the macronutrient composition of juçara, whereas feeding with 2 % juçara promotes injury owing to the composition of the fruit and overshadows the beneficial effects of polyphenols on glucose metabolism. However, higher juçara supplementation improves the inflammatory status targeted by the HC diet. An investigation of the action of juçara to identify its benefits and harmful effects as well as to define an optimal amount for cost-effective use awaits further study.

## Abbreviations

ANOVA: analysis of variance
AUC: area under the curve
BHT: 2,6-bis(1,1-dimethylethyl)-4-methylphenol
CAT: catalase
EPI: epididymal adipose tissue
FFA: Free fatty acids
GAE: gallic acid equivalent
GLUT-4: Glucose transporter type 4
HOMA-IR: Homeostasis Model Assessment
IL-10: Interleukin 10
IL-6: Interleukin 6
LPS: lipopolysaccharides
MES: mesenteric adipose tissue
OGTT: oral glucose tolerance test
RBP4: retinol binding protein 4
RET: retroperitoneal adipose tissue
TG: triacylglycerol
TNF-α: tumor necrosis factor alpha
